# *Clostridium Butyricum* CGMCC0313.1 Modulates Lipid Profile, Insulin Resistance and Colon Homeostasis in Obese Mice

**DOI:** 10.1371/journal.pone.0154373

**Published:** 2016-04-28

**Authors:** Haixiao Shang, Jia Sun, Yong Q. Chen

**Affiliations:** State Key Laboratory of Food Science and Technology, School of Food Science and Technology and Synergetic Innovation Center of Food Safety and Nutrition, Jiangnan University, Wuxi, P. R. China; Southern Illinois University School of Medicine, UNITED STATES

## Abstract

Obesity is associated with a cluster of metabolic disorders and systemic low-grade inflammation involving multiple organs. Recent findings have suggested that intestine is a key organ altered in response to high fat diet (HFD) feeding. Probiotics mainly *lactobacillus* strains have earlier been implicated in alleviating metabolic disorders. Here we aimed to examine the effects of a naturally occurring butyrate-producing probiotic *clostridium butyricum* CGMCC0313.1 (CB0313.1) in limiting the development of HFD-induced obesity. Mice treated with CB0313.1 exhibited reduced lipid accumulation in liver and serum, lower circulating insulin levels and improved glucose tolerance and insulin sensitivity. Furthermore, CB0313.1 administration reversed the HFD-induced colonic inflammation as evidenced by reduced tumor necrosis factor (TNF)-α level and increases the interleukin (IL)-10 and IL-22 levels in colon tissue. Additionally to colonic inflammation, CB0313.1 also reduced the colon permeability by upregulating the tight junction (TJ) proteins (claudin-1 and occludin) and contributed to a decreased circulating endotoxin level. In colon content, CB0313.1 administration restored the reduced production of butyrate and other short chain fatty acids (SCFAs) caused by HFD feeding. In adipose tissue, lower transcriptional levels of pro-inflammatory TNF-α, IL-6, IL-1β and monocyte chemotactic protein (MCP)-1 in adipose tissue were observed in CB0313.1-treated mice. Collectively, our data demonstrated that CB0313.1, targeting colon inflammation and permeability, ameliorated HFD-induced obesity, insulin resistance as well as adipose inflammation.

## Introduction

Obesity has reached epidemic proportions and acts as a major risk factor of many metabolic diseases including type 2 diabetes (T2D). Chronic low-grade inflammation of adipose tissue associated with increased production of inflammatory cytokines is a hallmark in the development of obesity [1]. Systemically enhanced cytokine productions interfere with insulin signaling pathway [2,3], resulting in systemic insulin resistance and the subsequent progression to T2D [4].

In addition to adipose tissues, intestine is another key site dysregulated during obesity[5]. Leaky gut mucosal barrier causes systemic endotoxin level to increase and further enhance chronic low-grade inflammation, thereby promoting the development of obesity [6]. A large body of evidence has shown that gut microbiota is altered during obesity and T2D. Additionally, butyrate-producing bacterias are decreased in patients with T2D compared with healthy controls [7,8]. Manipulations of resident microbes could influence whole-body metabolism by modulating the inflammation state and gut barrier function [9,10]. A recent study shows that IL22, a cytokine that maintains gut mucosal barrier integrity within the intestine, alleviates metabolic disorders and restores mucosal immunity [11]. In addition, 5-aminosalicyclic acid (5-ASA), a drug with anti-inflammatory properties and that acts locally in the colon, improves gut and adipose tissue inflammation as well as systemic insulin sensitivity [12]. These findings suggest that intestine is a novel target for therapeutic intervention in obesity and obesity-related insulin resistance.

CB0313.1 is a butyrate-producing, gram-positive bacteria and used as a probiotic for treating and preventing non-antimicrobial-induced diarrhea and irritable bowel syndrome. Butyrate is a short chain fatty acid (SCFA) together with others (acetate, propionate) produced in large amounts from dietary fibers after fermentation in the colon. Besides being a main energy substrate for colonic epithelium [13], butyrate plays a key role in maintaining gut immunological homeostasis [14]. Butyrate helps proliferation of intestinal mucosal cells [15], exerts anti-inflammatory effect in rat colitis [16], and suppresses nuclear factor(NF)κB activation in colonocytes [17]. Furthermore, butyrate produced in the intestine induces differentiation of colonic regulatory T cells [18] and promotes peripheral regulatory T cell generation[19]. Changes in proportions of CD4^+^ and Foxp3^+^ regulatory T cells have been shown in the colon of obese mice[12].

Here, we hypothesized that a *clostridium butyricum* probiotic may have beneficial effects on HFD-induced obesity and insulin resistance, by promoting SCFA production, improving colon barrier function as well as restoring colon immune homeostasis. To this end, we investigated the effects of CB0313.1 administration on HFD-induced body weight, metabolic markers and insulin sensitivity. Potential beneficial effects of CB0313.1 on colon homeostasis were investigated by evaluating colonic inflammation, production of SCFAs and colon permeability.

## Materials and Methods

### Mice and Experiment Design

4-week male C57BL/6 mice were used in this study. All experimental protocols were approved by the Animal Ethics Committee of Jiangnan University, China, and were performed according to the ethical guidelines of the European Community guidelines (Directive 2010/63/EU). Mice were maintained in a pathogen-free, temperature-controlled environment on a 12hr light and dark cycle at animal center of Jiangnan University. Mice were randomly divided into Normal diet (ND) group, HFD control group and HFD-CB group after acclimatization for 1 week. ND group and HFD group were administered with phosphate buffered saline (PBS). CB0313.1 is a spore-forming probiotic and stomach acid tolerant and it (suspended in PBS, 2×10^8^/day/mouse) was administered as soon as the HFD started.

### Metabolic chambers analysis

Respiratory exchange ratio (RER, the volume ratio of oxygen consumed versus CO2 exhaled) and spontaneous locomotor activity (counts) were measured using metabolic chambers (Columbus Instruments, Columbus, OH). Activity was detected using infrared light locomotion monitoring system. Mice were individually housed and acclimated to the chambers for 24 hour before experimental measurements.

### Histology

Fresh liver tissues were collected at 16 weeks of age after 12 weeks on each diet. Tissue was fixed in 10% formalin solution. Tissue slides were obtained through serial section cutting 5μm in thickness and stained with hematoxylin and eosin (H&E) as standard procedure.

### Lipids in serum and liver

Total cholesterol (TC), total triglyceride (TG) and low density lipoprotein-cholesterol (LDL-C) in serum were measured using an automatic biochemical analyzer (Mindray BS-480, Shenzhen, China). Free fatty acids (FFAs) in liver was determined by a colorimetric assay kit purchased from Nanjing Jiancheng Bioengineering Institute (Nanjing, China).

### Insulin Sensitivity

Glucose tolerance test (GTT) and insulin tolerance test (ITT) were performed at the end of this experiment (GTT at 10 week, ITT at 11week). Before the GTT test, animals were fasted overnight and 2g/ kg glucose was injected intraperitoneally. Before the ITT test, mice were fasted 4h and insulin (0.75U/kg) was injected intraperitoneally. Blood glucose levels were determined with an Accu-chek glucosemeter (Roche Diagnostics, Almere, The Netherlands) at stated time points.

### Insulin, cytokines and lipopolysaccharide (LPS) measurement

Insulin levels in serum were measured using an ELISA kit (Mouse Insulin ELISA, Mercodia, Sweden) as standard procedure. For colonic cytokines(TNF-α, IL-10 and IL-22), the colon tissue was cut and homogenized with saline(1:19, w/v), then the homogenate was centrifuged at 4°C for 10 min at 4000*g*, supernatant was used for ELISA analysis (Mouse TNF-α/IL-10/IL-22 ELISA Kit, Dobio Biology Technology, Shanghai). Serum LPS levels were determined by ELISA (Mouse LPS ELISA, Xinle, Shanghai).

### RNA isolation and qPCR

Total RNA was isolated from epididymal adipose tissue and colon using TRIzol (Invitrogen). Complementary DNA was prepared by reverse transcription of 2μg total RNA using a Reverse Transcription reagent kit (RT reagent Kit with gDNA Eraser RR047A, TaKaRa, Dalian). SYBR Green PCR reagents (BIO-RAD) were used to determine the mRNA levels. β-actin was used as a housekeeping gene. Calculations were made based on the comparative cycle threshold method (2^-△△Ct^). Primer sequences are given in Table 1.

**Table 1:** Primers used for qPCR analysis.

gene	forward	reverse
β-actin	5’-GGCTGTATTCCCCTCCATCG-3’	5’-CCAGTTGGTAACAATGCCATGT-3’
TNF-α	5’-AGGGTCTGGGCCATAGAACT-3’	5’-CCACCACGCTCTTCTGTCTAC-3’
IL-1β	5’-CTGAACTCAACTGTGAAATGC-3’	5’-TGATGTGCTGCTGCGAGA-3’
IL-6	5’-CTCTGCAAGAGACTTCCATCCAGT-3’	5’-GAAGTAGGGAAGGCCGTGG-3’
MCP-1	5’-CCCAATGAGTAGGCTGGAGA -3’	5’-TCTGGACCCATTCCTTCTTG-3’
Claudin-1	5’-GATGTGGATGGCTGTCATTG-3’	5’-CCTGGCCAAATTCATACCTG-3’
occludin	5’-CACACTTGCTTGGGACAGAG-3’	5’-TAGCCATAGCCTCCATAGCC-3’

### Colon content SCFAs quantification

Acetate, propionate and butyrate, present in the mice colon content were analyzed by the gas chromatography coupled mass spectrometer (GC-MS). Briefly, colon content samples (50mg) were first homogenized in 500μl of saturated NaCl solution. Thereafter, samples were acidified with 40μl 10% sulfuric acid. 1ml diethyl ether was added to the samples to extract SCFAs. Samples were then centrifuged at 14,000 *g* for 15 min at 4°C and supernatants were used for analysis. 1μL of supernatants were injected into Rtx-WAX capillary column (30m × 0.25mm×0.25μm, Bellefonte, PA, USA) installed on the GC-MS-QP2010 (Shimadzu, Japan). The initial oven temperature was 100°C and increased to 140°C at a rate of 7.5°C/min. The temperature further increased to 200°C at a rate of 60°C/ min and remained for 3 min. Helium was used as the carrier gas at a flow rate of 0.89 ml/min, and the column head pressure was 62.7 kPa. The injector was set at 240°C. The injection mode was split and the ratio was 10:1. For mass spectrometer, ion source temperature was 220°C, interface temperature was 250°C, and the scan range was from m/z 2 to 100. Real time analysis software GC-MS Postrun (GC-MS solution Version 2.72) was employed to calculate the concentrations of the acids.

An external standard method was employed to determine concentration of each SCFA.

### Western blotting

For mouse colon samples, RIPA (containing protease inhibitors, beyotime, Shanghai) was used to lyse the tissues. The homogenates were centrifuged at 4°C for 15 min at 5000*g* and the supernatant was used for western blot analysis. Equal amounts(50μg) of protein, as determined by a BCA protein assay (BCA Protein Assay Kit, beyotime, Shanghai) were separated using a polyacrylamide SDS–PAGE gel. After SDS–PAGE, proteins were transferred to a PVDF membrane following the manufacturer’s instructions. The membrane was blocked with 5% (wt/vol) skim milk in Tris-buffered saline (TBS)/Tween 20 for 1h at room temperature followed by incubation overnight at 4°C with GAPDH, occludin (Santa Cruz) and claudin-1(Life technology) antibodies diluted in 1% skim milk in TBS/Tween20. After overnight incubation, the membrane were incubated with horseradish peroxidase (HRP)-conjugated secondary antibodies at a dilution of 1:2000 in 5% (wt/vol) skim milk in TBS/Tween 20 for 2 h at room temperature and subsequently developed with western lightening plus ECL (PerkinElmer) according to the manufacturer’s instructions.

### Statistical analysis

All data are presented as mean ± SEM. The numbers of biological experiments were listed as n values and were specified in the figure legends. Difference was analyzed by unpaired Student’s t-test (GraphPad Prism 5). P<0.05 was considered statistically significant.

## Results

### CB0313.1 alleviates HFD-induced obesity

**Fig 1:**
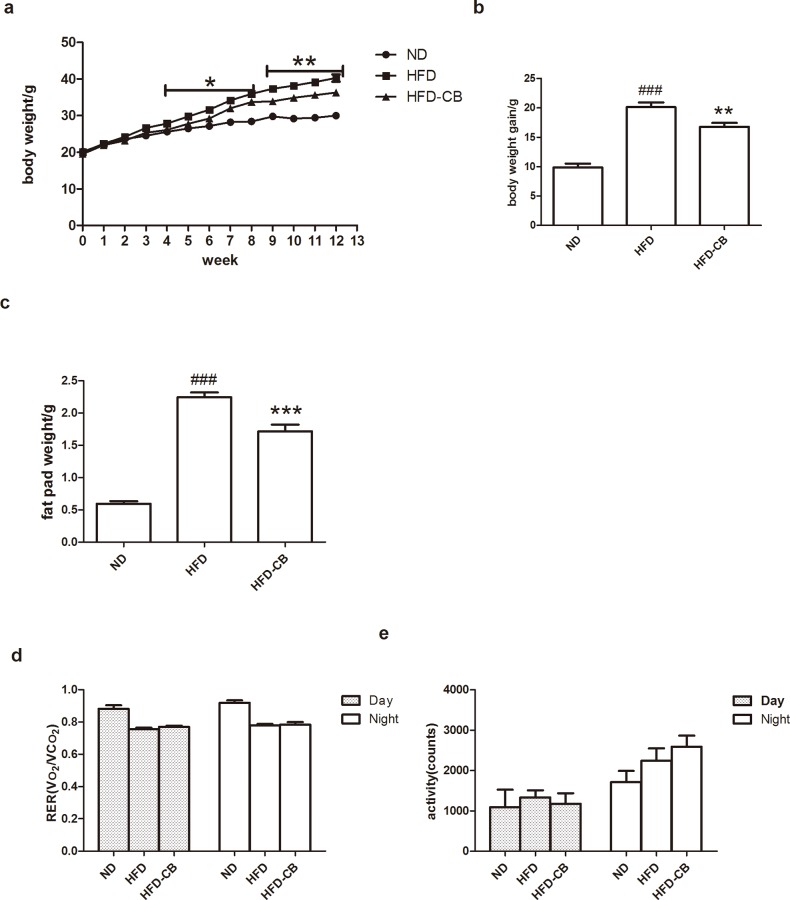
CB0313.1 on HFD induced obesity in mice and metabolic chamber analysis. Mice were fed ND, HFD, or HFD-CB for 12 weeks. (**a**) Body weight development and (**b**) body weight gain on ND, HFD or HFD received probiotic. (**c**) Epididymal fat pad was weighted. (**d**) Metabolic chambers analysis of RER and (**e**) spontaneous physical activity was shown as the counts of horizontal movement. For a-c, n = 15 in each group, for d and e, n = 4 per group. Error bars are shown as mean ± SEM. Significant difference between ND versus HFD are indicated as ###P<0.001, while significant difference between HFD and HFD-CB0313.1 are indicated as *P<0.05, **P<0.01, ***P<0.001.

To address the effects of CB0313.1 in obesity-related metabolic markers, the animals were fed with HFD (45% kcal derived from fat) or a control ND (10% kcal derived from fat) for 12 weeks. CB0313.1 treatment alleviated HFD-induced body weight gain compared with HFD control group. The difference became significant after 4-week HFD feeding (Fig 1A). After 12-week HFD feeding, total body weight gain was much lower in CB0313.1-treated group (Fig 1B). Similarly, the fat pad weights were reduced in CB0313.1-treated group compared to HFD group (Fig 1C).

The effect of CB0313.1 on metabolic markers including RER and spontaneous locomotor was then examined using metabolic chambers. ND group showed higher RER compared to HFD fed mice, suggesting a lower respiratory exchange ratio in obese mice. CB0313.1 administration did not affect RER or spontaneous locomotor activities in HFD-fed groups (Fig 1D and 1E).

### CB0313.1 lowers lipid levels in liver and serum

**Fig 2:**
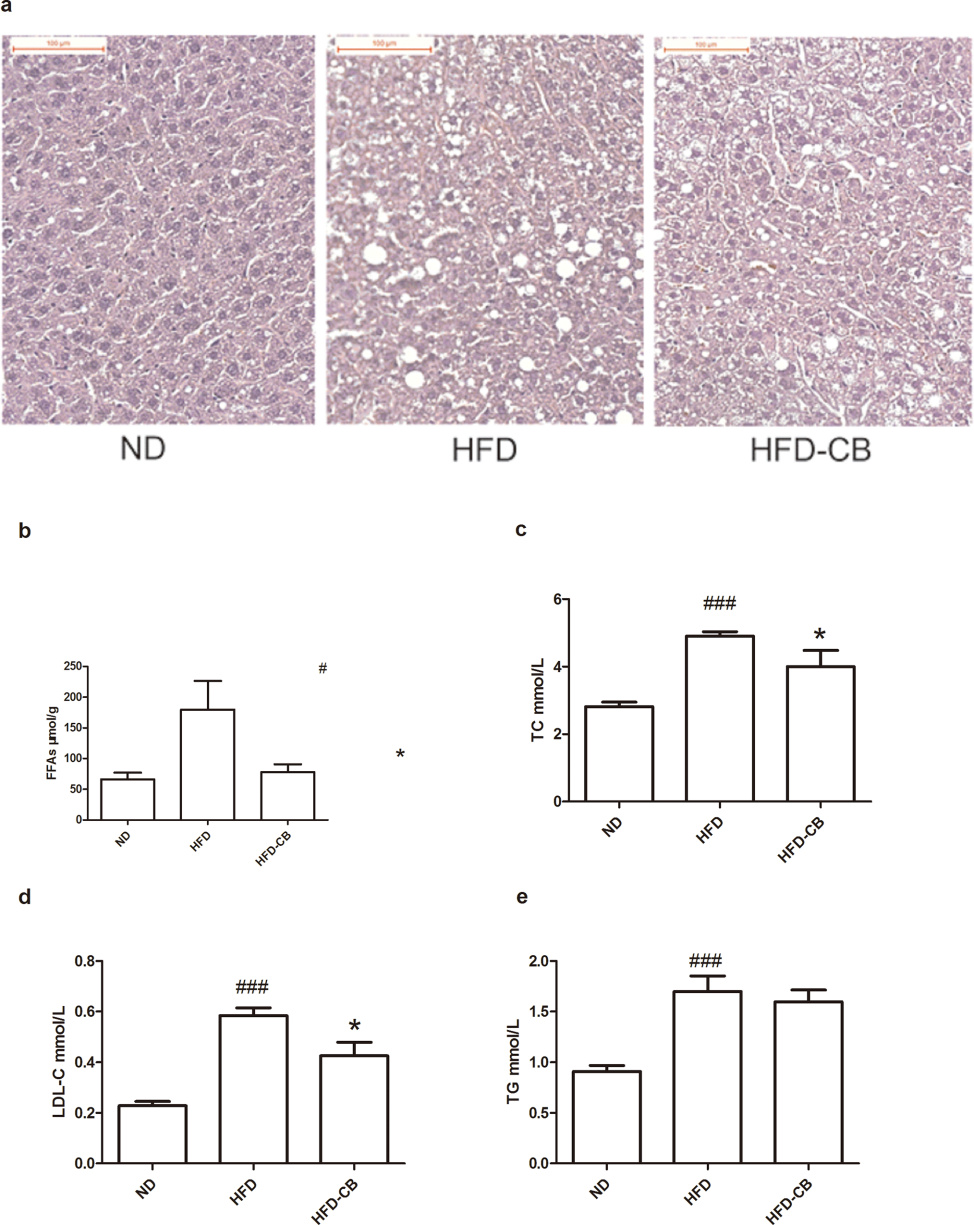
CB0313.1 on lipids in liver and serum. Mice were fed ND, HFD, or HFD-CB for 12 weeks (**a**) Haematoxylin and eosin (H&E) staining of liver sections (scale bar, 100μm), (**b**) FFAs level in liver was measured (n = 9 per group), (**c**) TC, TG, LDL-C in serum (n = 7–9 per group) were measured. Error bars are shown as mean ±SEM. Significant difference between ND versus HFD are indicated as #P<0.05, ###P<0.001, while significant difference between HFD and HFD-CB are indicated as *P<0.05.

In the liver, CB0313.1 reduced hepatic steatosis induced by HFD. As visualized by H&E staining, CB0313.1 intake decreased accumulation of lipid droplets caused by HFD feeding (Fig 2A). This observation was in line with liver FFAs in that HFD-CB group showed significant lower FFAs compared with HFD group and the difference between HFD-CB and ND is non-significant (Fig 2B). In the serum, TC and LDL-C levels were lower in CB0313.1-treated animals compared with HFD feeding controls while there was no difference in TG level (Fig 2C).

### CB0313.1 improves insulin sensitivity in HFD-induced obesity mice

**Fig 3:**
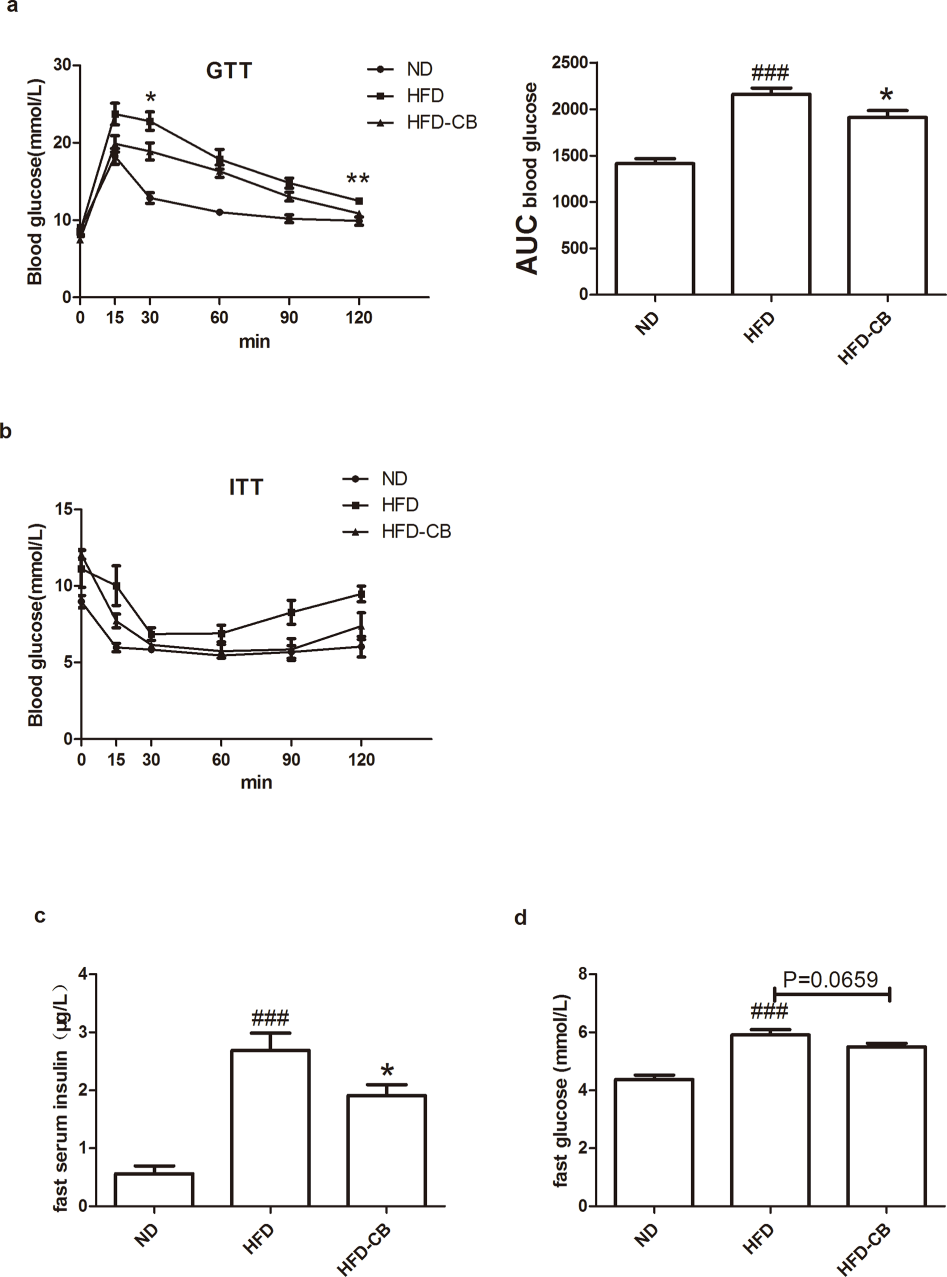
CB0313.1 on insulin sensitivity. (**a**) Intraperitoneal GTT on each diet and each treatment (left) and area under curve (AUC), GTT was performed at 10th weeks (n = 5). (**b**) ITT on ND, HFD or HDF-CB (left) and AUC, ITT was performed at 11th week (n = 5). (**c**) Fast serum insulin levels on ND, HFD or HFD-CB (12 week, n = 9) and (**d**) Fast blood glucose (12week, n = 15). Error bars are shown as mean ± SEM. Significant difference between ND versus HFD are indicated as #P<0.05, ##P<0.01, ###P<0.001, while significant difference between HFD and HFD-CB are indicated as *P<0.05.

The beneficial effects of CB0313.1 on obesity development suggest that CB0313.1 may protect mice from HFD-induced insulin resistance. To test this, we performed GTT and ITT. The data revealed that CB0313.1-treated mice had improved glucose tolerance (Fig 3A) and relatively better insulin sensitivity (Fig 3B) compared with HFD controls. In addition, CB0313.1 treatment showed preserved fasting insulin levels in the serum, similar tendency was observed in blood glucose while the result was not significant (Fig 3C and 3D).

### CB0313.1 suppresses colon inflammation and increases SCFAs production

**Fig 4:**
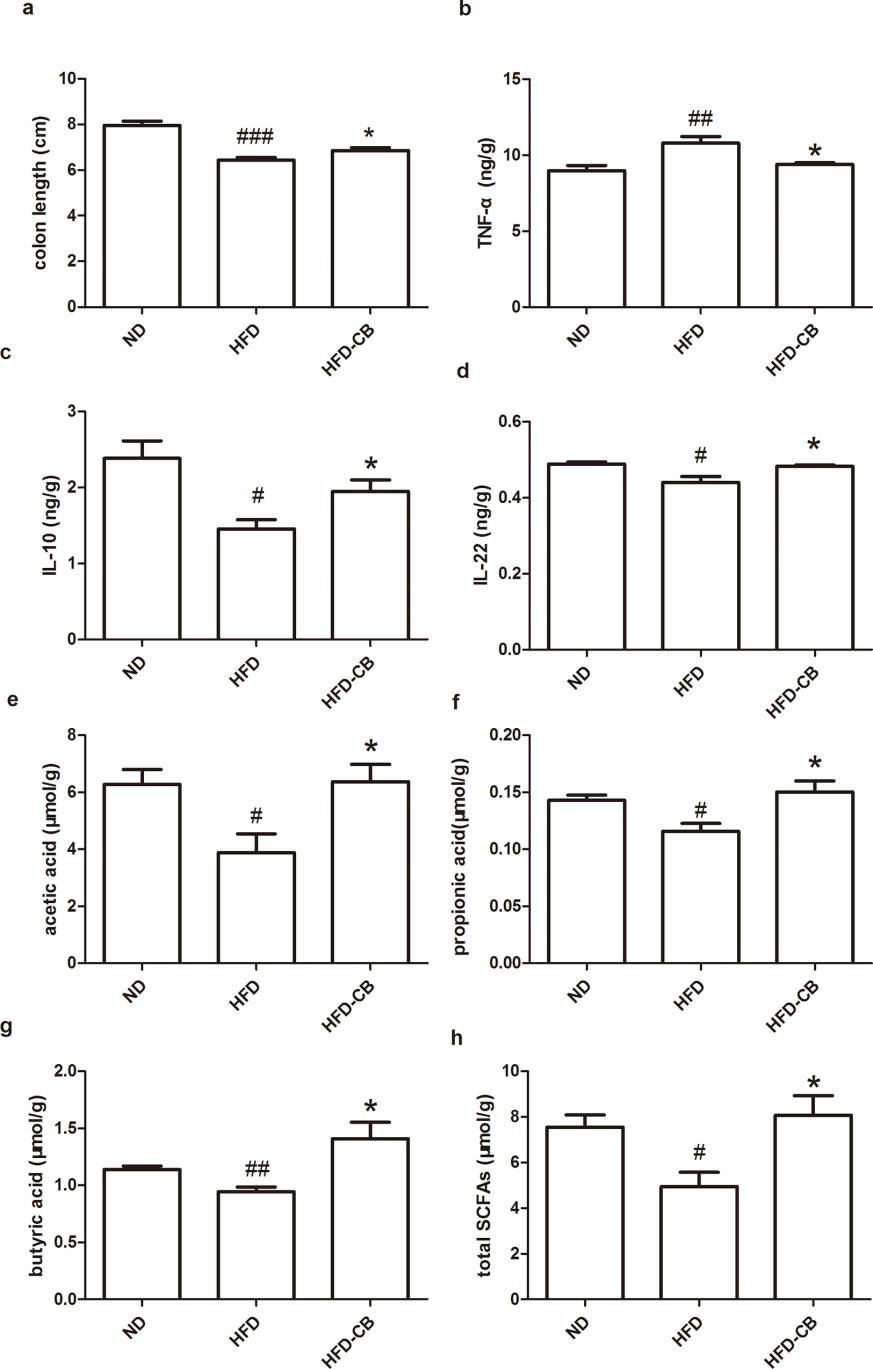
CB0313.1 on HFD-induced low-grade colitis and SCFA production. (**a**) Colon length was measured (n = 15). (**b**-**d**) Concentrations of cytokines in homogenized supernatant of colon tissue were measured by ELISA (n = 5). (**e**-**h**) SCFAs (acetate, propionate and butyrate) and total SCFAs in colon content were determined by GC-MS (n = 6). Error bars are shown as mean ± SEM. Significant difference between ND versus HFD are indicated as #P<0.05, ##P<0.01, ###P<0.001, while significant difference between HFD and HFD-CB are indicated as *P<0.05.

Recent studies have indicated low-grade colitis in HFD feeding mice [12,20]. Next, we investigated whether this butyrate-producing probiotic has any regulatory effect on HFD-induced colon inflammatory status. HFD-fed mice exhibited shortened colon length after 12 weeks of feeding and CB0313.1 intake protected this shortening (Fig 4A). The pro-inflammatory cytokine, TNF-α and anti-inflammatory (IL-10) cytokine were measured to assess the regulatory effect of CB0313.1 on inflammation. CB0313.1-treated mice showed a lower TNF-α level as well as a higher IL-10 level compared with HFD control animals (Fig 4B and 4C). IL-22, which helps maintaining the intestine integrity and elicit antimicrobial immunity [21,22], was reduced in HFD feeding group and restored by CB0313.1 intake (Fig 4D). There was no significant difference between ND and HFD-CB mice in TNF-α, IL-10 or IL-22 levels.

SCFAs produced in colon have demonstrated multiple beneficial metabolic effects. Therefore, we studied the effect of CB0313.1 on SCFA production. The data showed that CB0313.1 restored HFD-induced low SCFAs production and HFD-CB group showed no significant difference in SCFA concentrations in colon content compared with ND group (Fig 4E–4H).

### CB0313.1 improves colon permeability and ameliorates adipose inflammation

**Fig 5:**
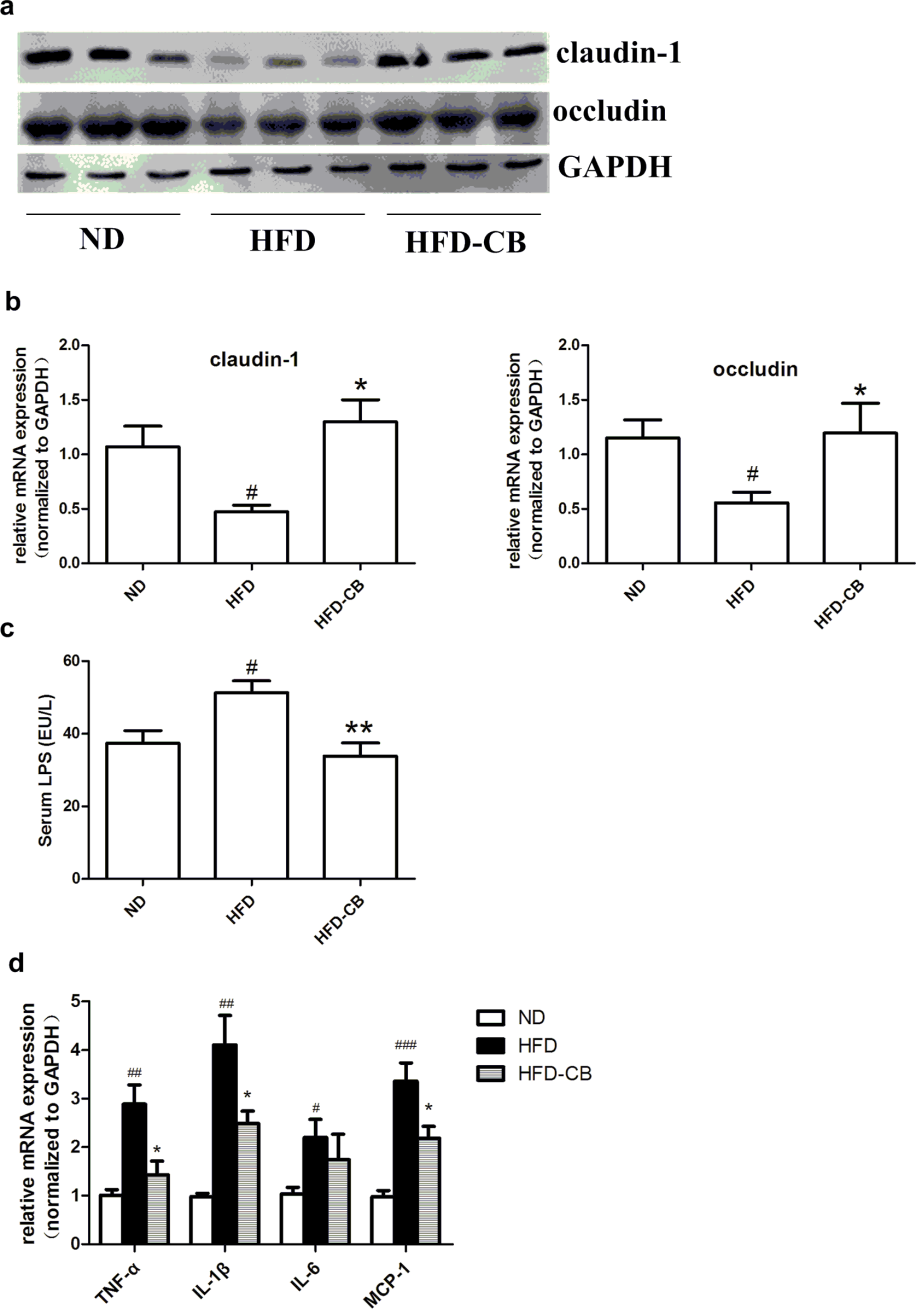
CB0313.1 on TJ proteins expression, serum LPS and epididymal adipose inflammation. (a) Western blot analysis of TJ proteins, claudin-1, occludin and GAPDH as housekeeping protein (n = 3), (**b**) TJ proteins (claudin-1 and occludin) mRNA expression in mouse colon were determined by RT-qPCR (n = 5). (**c**) Concentration of LPS in serum were measured by ELISA (n = 5). (**d**) Epididymal adipose inflammation were determined by the transcriptional levels of pro-inflammatory gene (TNF-α, IL-1β, IL-6 and MCP-1, n = 4). Significant difference between ND versus HFD are indicated as #P<0.05, ##P<0.01, ###P<0.001, while significant difference between HFD and HFD-CB are indicated as *P<0.05, **P<0.01.

TJ proteins regulate the permeability of intestinal barrier [23]. Since both HFD-induced obesity and colon inflammation are accompanied by impaired colon epithelial barrier and colon permeability, we investigated the effect of CB0313.1 on colon permeability by measuring expression of TJ proteins and serum LPS level. Western blot analysis and RT-qPCR analysis showed that CB0313.1 upregulated the expression of claudin-1 and occludin at both the protein and mRNA levels (Fig 5A and 5B). Consistently, serum LPS level in CB0313.1 treatment group was decreased (Fig 5C). Between ND animals and HFD-CB animals the difference in TJ protein mRNA and serum LPS is non-significant.

Enhanced LPS leakage from the gut is a well-established mechanism that causes systemic low-grade inflammation and contributes to insulin resistance [1]. Since CB0313.1 improved colon permeability and reduced serum LPS level, we next investigated effects of CB0313.1 on the regulation of epididymal adipose inflammation. Transcriptional levels MCP-1 and cytokine (TNF-α, IL-1β and IL-6) were measured using qPCR. CB0313.1 administration significantly reduced the transcriptional levels of TNF-α, IL-1β and MCP-1 (Fig 5C), suggesting that CB0313.1 ameliorated inflammation in adipose tissue induced by HFD.

## Discussion

In this study, we proved that treatment with the butyrate-producing probiotic CB0313.1 protected against the development of HFD-induced obesity and improved obesity-related insulin resistance. First, these conclusions are based on the observation that mice treated with CB0313.1 showed a reduction in the body weight development and fat pad weight in response to HFD feeding in comparison with mice fed the same diet. Second, CB0313.1 significantly lowered the lipid accumulation and FFAs level in the liver compared with HFD controls, the lipid profiles in serum showed the same tendencies. Third, CB0313.1 improved the insulin sensitivity which is evidenced by the lower blood glucose when challenged with glucose and insulin at stated time points. Fourth, CB0313.1-treated mice showed a preserved fast blood glucose and fast serum insulin. Taken together, CB0313.1 protected the progression of obesity and insulin resistance.

Butyrate produced by fermentation of dietary fibers exerts various protective activities in colon. Addition of sodium butyrate (5%) in HFD significantly improved insulin resistance induced by HFD as well as enhanced energy expenditure [24]. In this study, we observed that CB0313.1 intake limited the development of obesity and enhanced butyrate production in colon. However, there was no significant difference on RER and spontaneous locomotor activity in CB0313.1-treated mice compared with HFD controls. This suggests that CB0313.1, different from sodium butyrate, did not affect energy metabolism, likely due to its absorption and effects largely exerted locally in the colon.

Low-grade colitis is closely related with HFD-induced obesity. It has been previously demonstrated that HFD induces the expression of several pro-inflammatory cytokines (TNF-α, IL-1β and IL6) as well as activation of NFκB in colon [20]. A recent report investigated resident immune cells in the intestines and showed a shift in immune cell populations towards inflammatory phenotypes in mice fed with HFD [12]. In our study, similar inflammatory responses were observed in HFD-fed mice compared to ND controls. As a naturally occurring probiotic, CB0313.1 suppressed HFD-induced colonic inflammation as evidenced by improved colon length and a modulatory cytokine profile compared with HFD controls. Furthermore, CB0313.1 increased colon IL-22 expression, which has a beneficial effect on colon barrier integrity and metabolic disorders [11].

SCFAs exert prominent roles in the intestinal immune homeostasis. In addition, they may act as signaling molecules by binding with G protein-coupled receptor 41 and affect host adiposity [25]. Recent studies show that SCFAs produced in intestine induce intestinal gluconeogenesis gene expression which promotes metabolic benefits [26]. Propionate in intestine has an effect on appetite regulation and thus affects the whole body metabolism [27]. Prebiotics such as fructo-oligosaccharides (FOS) and pectin that could be fermented by gut microbiota to form SCFAs afforded beneficial effects on the host [26,28]. In our study, GC/MS and an external standard method were employed to detect acetate, propionate and butyrate in colon content. Results showed that HFD significantly reduced the production of acetate, propionate and butyrate in colon compared with ND controls after 12 weeks of HFD feeding. This was in accordance with an earlier observation [29]. In CB0313.1-treated mice, the productions of SCFAs were restored. Given the beneficial effects of butyrate on colonic epithelia [13] and intestinal immune modulation [14], our data suggest that CB0313.1 has a direct effect in the colon and contributes toits homeostasis state in gut microenvironment by balancing SCFA production

Colon permeability is controlled by TJ proteins and colon leakage is closely related with the development of obesity [30]. Previous studies reported that HFD accelerates weight loss in dextran sulfate sodium-induced colitis in mice [31] and obese mice show reduced survival rate during *Citrobacter rodentium* infection due to poor intestine integrity. These data suggest that HFD-induced obesity is closely associated with impaired intestinal barrier function. In addition, it has been reported that some probiotics and prebiotics could up-regulate TJ protein expression and restore colon barrier integrity [32]. In our study, CB 0313.1 significantly restored obesity-induced down-regulation of claudin-1 and occludin at both transcriptional and protein levels. Colon derived LPS is shown to contribute to systemic inflammation and development [1]. Accordingly, we demonstrated that CB0313.1 decreased the serum LPS level and suppressed the adipose inflammation.

**Fig 6:**
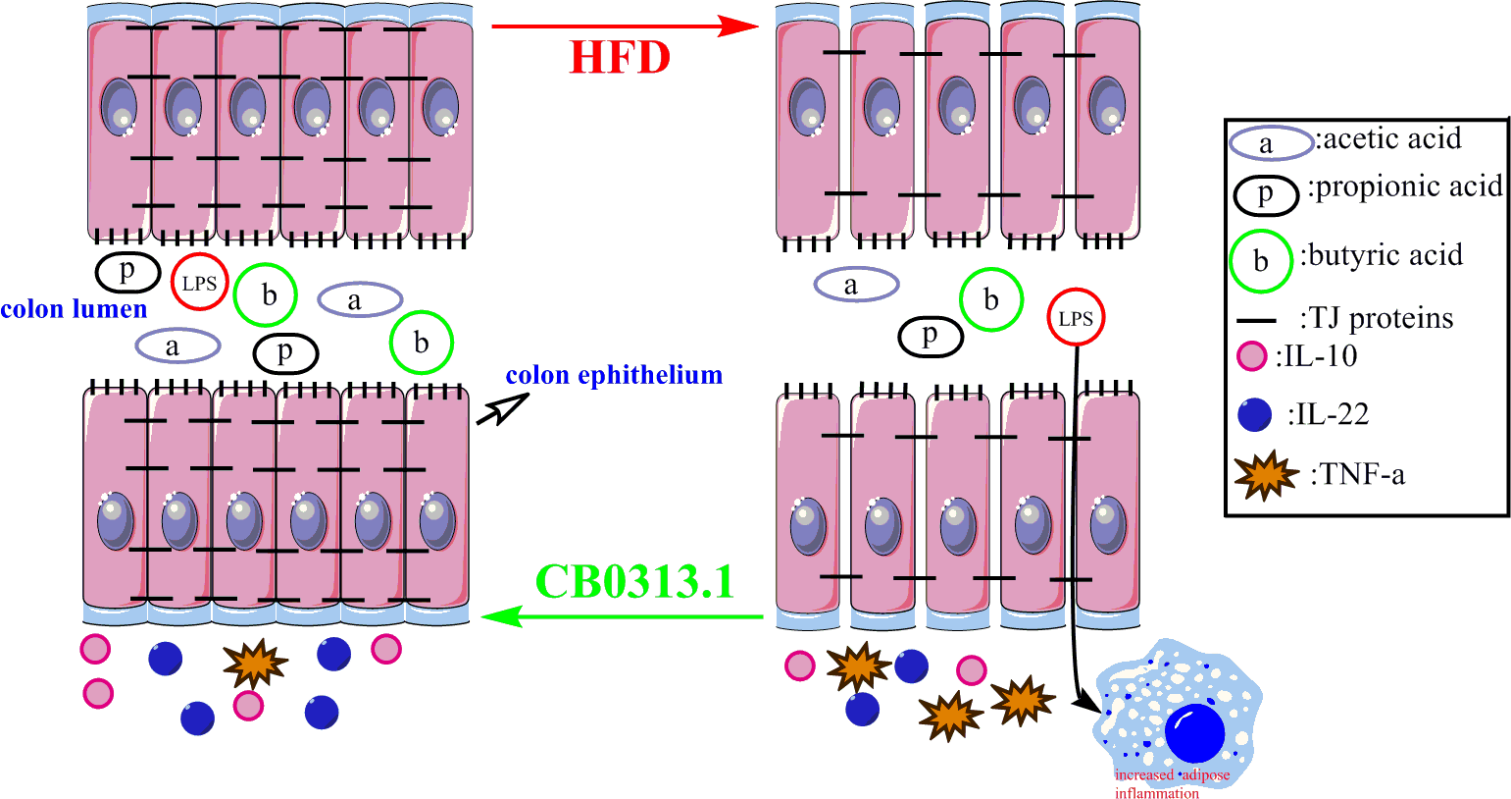
Summary of HFD on colon environment and the effect of CB0313.1. HFD induces colonic inflammation as evidenced by increased TNF-α and reduced IL-10. HFD increases the colon permeability through down-regulating the expression of TJ proteins and contribute to adipose inflammation. HFD also decreases the SCFAs levels. CB0313.1 reverses the HFD-induced alterations by maintaining the immune hemostasis, improving the colon permeability and producing beneficial SCFAs.

Taken together, CB0313.1 suppressed HFD-induced low-grade colitis as well as increased the SCFAs production in colon. Additionally, CB0313.1 restored the impaired colon permeability, reduced the circulating LPS and further ameliorated adipose inflammation (Fig 6).

Prebiotics such as pectin and FOS promote SCFAs production by gut microbiota [26,29]. A recent study used microarray analysis has demonstrated that symbiotic administration suppressed colon epithelial inflammation [33]. It remains to be investigated whether CB0313.1 together with prebiotic may have a synergistic effect on HFD-induced obesity.

Obesity is related with a cluster of alterations in colon including microbiota dysbiosis, enhanced inflammation, poor barrier function and lower SCFAs production. The current study has demonstrated that the butyrate-producing probiotic, CB0313.1, counteracts the detrimental effects of HFD feeding on body weight gain and insulin resistance, and helps maintaining gut immune and barrier homeostasis, thereby contributing to modulation of systemic adipose inflammation. CB0313.1 represents a promising probiotic agent for obesity and associated metabolic disorders.
